# Heat Transfer Enhancement of Small-Diameter Two-Phase Closed Thermosyphon Using Cellulose Nanofiber and Hydrophilic Surface Modification

**DOI:** 10.3390/nano11030647

**Published:** 2021-03-06

**Authors:** Dongnyeok Choi, Gyosik Jun, Woonbong Hwang, Kwon-Yeong Lee

**Affiliations:** 1 Department of Mechanical and Control Engineering, Handong Global University, 558 Handong-Ro, Heunghae-eup, Buk-gu, Pohang-si 37554, Korea; cdn4400@gmail.com; 2 Department of Mechanical Engineering, Pohang University of Science and Technology (POSTECH), 77 Cheongam-ro, Nam-gu, Pohang-si 37673, Korea; ksjeon8385@postech.ac.kr

**Keywords:** two-phase closed thermosyphon, confinement effect, geyser phenomenon, cellulose nanofluid, hydrophilic surface modification

## Abstract

In this study, we observed the Geyser phenomenon that occurs in a small-diameter two-phase closed thermosyphon (confinement number of 0.245). This phenomenon interferes with the natural circulation of the internal working fluid and increases the thermal resistance of the system. This study attempts to improve the thermal performance of the system using cellulose nanofiber as the working fluid and hydrophilic surface modification at the inner surface of the evaporator section. As a result, the total thermal resistance showed average reduction rates of 47.51%, 36.69%, and 22.56% at filling ratios of 0.25, 0.5, and 0.75, respectively.

## 1. Introduction

Due to the depletion of petroleum energy and distrust of the stability of nuclear energy, the need for eco-friendly energy is emerging. Unlike solar photovoltaic energy systems that convert light energy into electrical energy, solar thermal energy cannot collect heat high enough to run a turbine that collects electricity, but it can make hot water for use. For hot water production, a medium is needed to collect solar heat and deliver it to water. Two-phase closed thermosyphon (TPCT) is the most frequently used heat transfer medium for solar heat collection. As TPCT uses the latent heat of boiling and condensation of working fluid, its thermal resistance is low. Therefore, the amount of heat transfer is significant despite an insignificant temperature difference [1]. However, due to the operation principle, the uprising vapor and down-falling condensate collide. If the channel diameter of the TPCT is insufficiently small, stagnation occurs in the middle of the channel, thereby reducing the heat transfer performance significantly. This phenomenon is known as the confinement effect [2,3,4]. By reducing the diameter of the TPCT and installing more TPCT, it is possible to collect a larger amount of solar heat in the same area of space. The confinement number (Co), which is the relevant dimensionless number, is the ratio of the capillary length (or Laplace length, $\lambda_{c}$) of the working fluid to the channel diameter ($D_{i}$), as shown in Equation (1) below.

(1) $$\mathrm{Co}\;=\;\frac{\lambda_{C}}{D_{i}}\;=\;\frac{\sqrt{\frac{\sigma }{\left(\rho_{l}-\rho_{v}\right)g}}}{D_{i}}$$

where σ, $\rho_{l}$, $\rho_{v}$, and g refer to surface tension, density of water, density of vapor, and acceleration of gravity, respectively.

**Figure 1:**
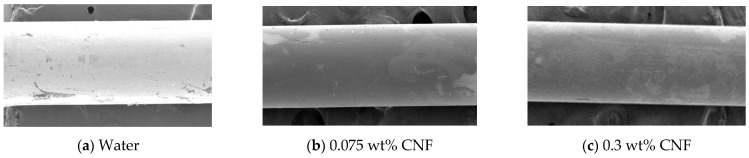
Scanning electron microscopy (SEM) image of wire after the experiment.

Previous studies showed that Co less than 0.12 implies a conventional TPCT that does not exhibit the confinement effect. In a TPCT with 0.12 < Co < 0.34, the confinement effect occurs and fails repeatedly, which is known as the Geyser phenomenon [2,3,4,5,6,7]. In a TPCT with Co > 0.34, the stagnation of the working fluid does not diminish but continues; this is known as the entrance limit [8]. When this phenomenon occurs, the heat of the evaporator is not transferred effectively and dry out occurs rapidly; therefore, it can no longer be a heat transfer medium [8]. Choi and Lee [9] designed a TPCT experiment apparatus with a channel that satisfied Co = 0.245 using deionized (DI) water as the working fluid. They observed that the temperature at the evaporator surface oscillated regularly with a large amplitude in the steady state, thereby confirming the Geyser phenomenon. In addition, cellulose nanofiber (CNF) fluid was used instead of DI water as the working fluid, and it was confirmed that the critical heat flux (CHF) and boiling heat transfer coefficient (BHTC) of the system improved. Among boiling studies involving nanofluids, most studies reported improved CHF [10,11,12,13,14]. However, various results of the BHTC have been reported. Some studies reported the “deposition” of nanoparticles on the heater surface, which improved the BHTC [10,11]. Other studies revealed a thick “coating” of nanoparticles on the heater surface, resulting in an increase in thermal resistance and a decrease in the BHTC [12,15]. Other studies showed the change in the BHTC of the nanofluid case was slight compared to that of water [13,14]. In addition, Suriyawong and Wongwises studied pool boiling using ${\mathrm{TiO}}_{2}$ nanofluid and reported that its BHTC was higher and lower than that of water in low and high concentration nanofluids, respectively [16]. It was discovered that the high conductivity of nanoparticles enhanced the BHTC at the low concentration; however, the higher the concentration, the thicker the coating of nanoparticles on the heater surface, resulting in a higher thermal resistance. In contrast, in a wire pool boiling study by Choi and Lee [9] using CNF fluid, the BHTC was lower than that of water at low concentrations (0.07, 0.1 wt%), but higher at high concentrations (0.3, 0.5 wt.%). Figure 1 shows the scanning electron microscopy (SEM) images of the wire surface after each experiment [9]. As shown, an extremely thin coating had formed on the wire surface. However, the coating thickness did not change by concentration. This was due to the characteristics of CNF, in which the particles do not aggregate with each other [17]. At low concentrations, the thermal resistance increased with the thin coating. As the concentration increased, the thickness of the coating remained the same, but the bubble frequency generated on the wire surface accelerated [9] and heat transfer was enhanced. 

In this study, to enhance the heat transfer performance of the TPCT of Co = 0.245, hydrophilic surface modification was additionally applied to the evaporator inner surface in addition to the CNF fluid. Previous studies have attempted to improve the heat transfer coefficient of conventional TPCT (Co < 0.11) by reducing the wettability of the condenser inner surface [18,19,20] or improving the wettability of the evaporator’s inner surface [18,19,21]. In this study, because the focus was on improving the boiling heat transfer performance, surface modification was applied only to the evaporator’s inner surface. In addition, the large Reynolds number of the cooling water flowing through the cooling jacket, which served as a heat sink for the condenser section, was applied. It had a sufficient cooling capacity, resulting in the temperature change on the condenser inner surface being insufficient during the TPCT operation.

## 2. Materials and Methods

### 2.1. Cellulose Nanofiber (CNF)

CNF is an eco-friendly organic nanofiber (purchased from A&Poly) and is manufactured using commercial coffee filters based on the method proposed by the Isogai group [17]. The measured properties of the 0.5 wt% CNF fluid were as follows (Table 1). It had aa neutral acidity of pH 7.42 and electrical conductivity of 1.658 mS/cm, which was much higher than that of DI water at 0.0005 mS/cm. Its density was 0.9984 $g/{\mathrm{cm}}^{3}$, which did not indicate a significant difference from that of DI water of 0.9970 $g/{\mathrm{cm}}^{2}$; however, the viscosity was 0.0089 $\mathrm{Ns}/m^{2}$, which was slightly larger than that of DI water at 0.00089 $\mathrm{Ns}/m^{2}$. In existing inorganic nanofluids, nanoparticles aggregate and settle over a significant period of time, which is disadvantageous [22,23]. CNF has an extremely low zeta potential (−92.83 mV at 0.5 wt%) and is negatively charged in solution; therefore, it affords the advantage of minimal aggregation or sedimentation between particles [17]. 

As the CNF supplied by the manufacturer was in the form of a gel with a concentration of 2 wt%, the appropriate amount of DI water was added to obtain the desired concentration of CNF fluid. For an effective dispersion of the CNF in the added water, an ultrasonic homogenizer (HUH-606, Hantech) was used to perform ultrasonic dispersion for 30 min. The density and surface tension required for the calculation of Co were assumed to be the same as those of DI water. In fact, the density of the 0.5 wt% CNF fluid and DI water exhibited a difference error of 0.14%. Although the surface tension could not be measured because of the absence of measuring equipment, it was assumed that the error (e.g., of the density) with DI water would not be large because 0.5 wt% is an extremely low concentration.

### 2.2. Hydrophilic Surface Modification

**Figure 2:**
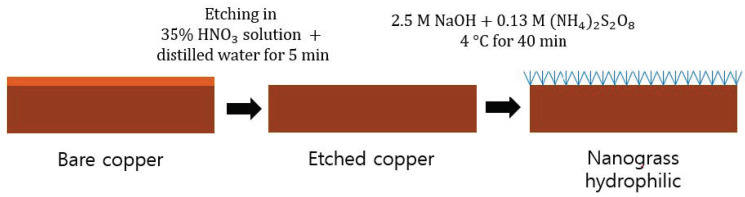
Fabrication process of the nanograss hydrophilic copper tube.

The chemical surface treatment process shown in Figure 2 was performed to apply hydrophilic surface confinement to the evaporator channel. The surface treatment process is as follows. First, to surface-modify only the inner surface of the copper tube, an acid-resistant tape was applied to the outer surface of the copper tube to prevent contact with the chemicals. Subsequently, the tube was etched by immersing it in a solution in which a 70% nitric acid (${\mathrm{HNO}}_{3}$) solution and distilled water were mixed at a volume ratio of 1:1 for approximately 1 min at room temperature. The etched tube was washed thoroughly with distilled water so that no chemical solution remained on the surface. Subsequently, the tube was immersed in a mixture of 2.5 M sodium hydroxide (NaOH) solution and 0.13 M ammonium persulfate $({\left({\mathrm{NH}}_{4}\right)}_{2}S_{2}O_{8}$) solution for 40 min, and the solution was maintained at a temperature of 4 °C in a refrigerator before and during the immersion. After the surface treatment, the tube was washed with distilled water, and the acid-resistant tape on the outer surface of the tube was removed. After washing the inside/outside of the tube again, it was dried in an oven at 60 °C for more than 1 h to completely remove the moisture.

**Figure 3:**
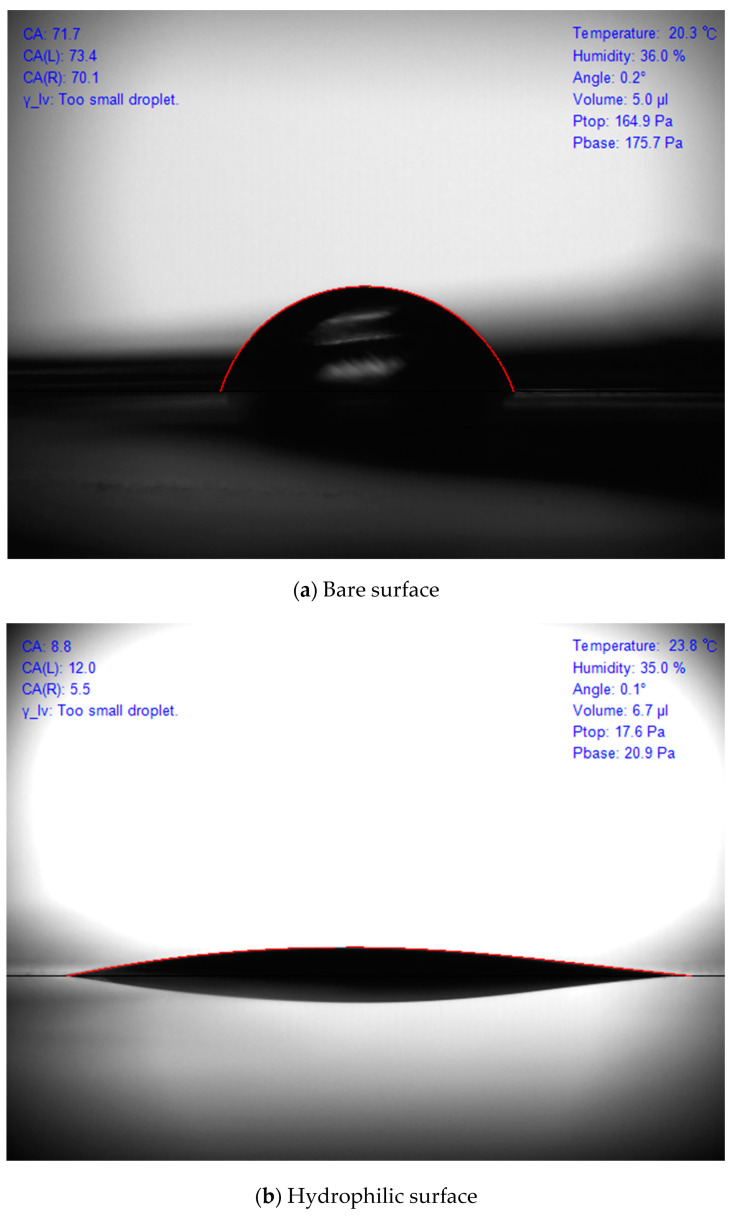
Surface contact angle of the copper surface.

**Figure 4:**
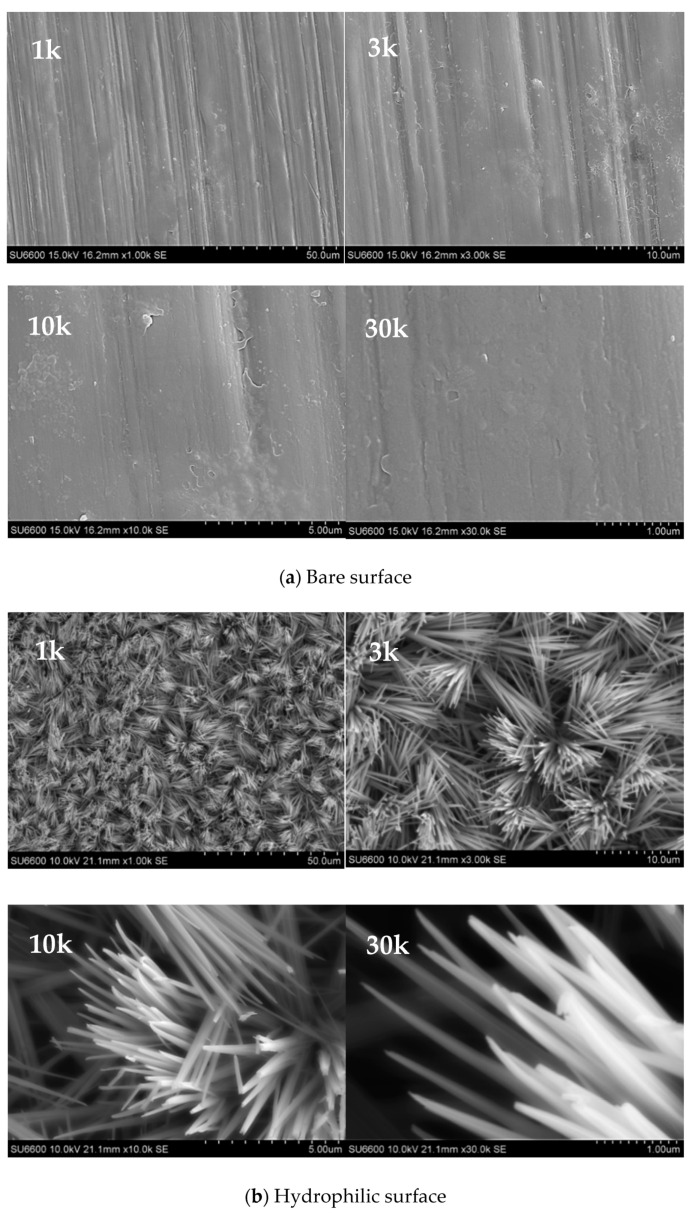
SEM image of the copper surface.

The contact angle of the surface droplets and the microstructure of the surface were photographed to verify the wettability of the surface-modified specimen. As surface modification was applied to the inner surface of the small-diameter tube, it was difficult to photograph the surface. For a smooth image capture, a plate specimen (measuring 50 mm (length) × 5 mm (width) × 1 mm (thickness)) produced by applying the same surface treatment method to the same material was used. The contact angle of the water droplet was measured using a measuring device (Smartdrop, FEMTOFAB) by depositing 5 μL of water droplets on the plate specimen. As shown in Figure 3, the hydrophilic surface had a small contact angle of 8.8°, which was significantly smaller than the contact angle of the convex droplet on a bare surface (71.7°). The nanostructure of the specimen surface was photographed using SEM (JEOL 7401F, JEOL) (Figure 4). As shown, unlike the smooth bare copper plate, a nanoscale structure was formed on the modified surface. Copper hydroxide has rod-like structures that are several micrometers in length and 1000–2000 nm in diameter, as shown in the SEM image.

### 2.3. Experimental Apparatus

**Figure 5:**
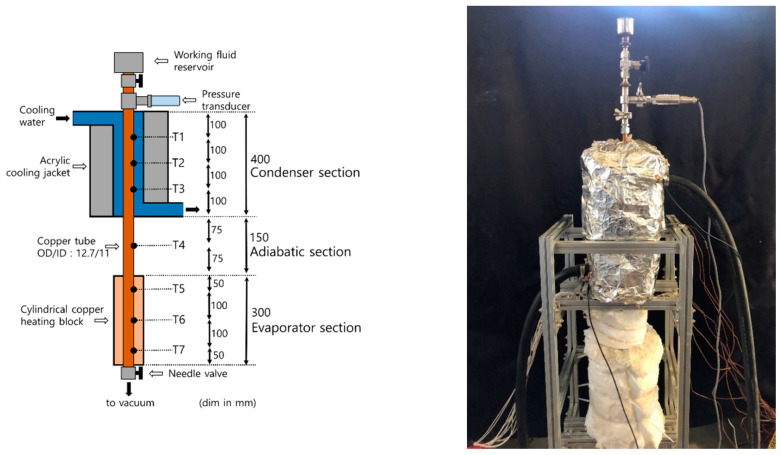
Experimental apparatus [9].

**Figure 6:**
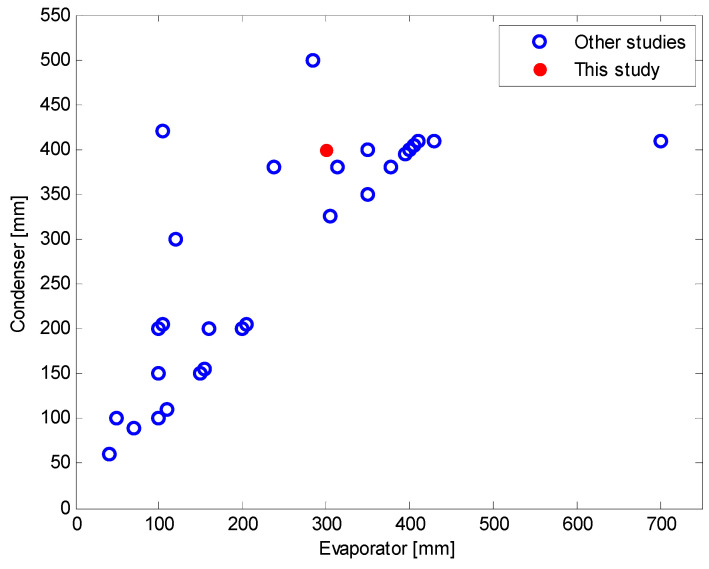
Length of the evaporator and condenser of two-phase closed thermosyphon (TPCT) in previous studies.

Figure 5 [9] shows a schematic illustration and photograph of the TPCT used in the experiment. The dimensions of the TPCT are as follows. When the working fluid was DI water, the inner diameter of the channel to satisfy Co = 0.245 was 11 mm. The lengths of the evaporator, adiabatic, and condenser sections were 300, 150, and 400 mm, respectively. Due to the heat-resisting limitation of the copper heater, an input power of 0–800 W was applied. The inner surface area of the evaporator section was set so that the heat flux was similar to the average input heat flux range (0–80 $\mathrm{kW}/m^{2}$) of previous studies. As the diameter of the channel can be determined by considering Co, the length of the evaporator section was set by considering the heat flux range; hence, it was set to 300 mm. Figure 6 shows a comparison of the lengths of the evaporator and condenser sections of this study and those of previous studies. As shown, the length of the evaporator section set in this study (300 mm) was an approximate average value compared with those of previous studies. To maximize the condensation performance and maintain the temperature of the condenser section through the rapid condensation of steam, the condenser section was set to the maximum length that did not deviate significantly from those of other studies. The length of the adiabatic section was the average value obtained from previous studies.

The material of the channel as well as that of the heater that supplied heat to the evaporator channel were copper. The heater was connected to a power supply (N8953A, Keysight) to power the copper heater. The contact thermal resistance between the evaporator channel and copper heater was minimized by filling aluminum nitride powder of thermal conductivity 150 W/mK. The condenser was cooled by the cooling water flowing through the cooling jacket. The cooling jacket, which was made of acrylic, was supplied with cooling water at a specified temperature and flow rate from the connected chiller (GR-C-00050A, Busung). Subsequently, 3, 2, and 3 k-type thermocouples (Omega) were attached to the evaporator, adiabatic, and condenser section channel outer surface, respectively. All of these thermocouples were precisely calibrated to exhibit an error of ±0.15 °C. The temperature data of the thermocouples were collected at 1 s intervals using a data acquisition system (34970A, Keysight) connected to the thermocouples. At the upper end of the condenser section, a pressure transmitter (PSHJ1000TCTJ) with an error rate of ±0.15% was attached to measure the internal vacuum pressure. A ceramic fiber insulator with a thermal conductivity of 0.049 W/mK (at 200 °C) and thickness of 10, 5, and 5 cm was applied to the evaporator, adiabatic, and condenser sections, respectively. As the outer surface temperature of the insulation material showed an error of less than 2 °C from the room temperature during the actual experiment, we could assume that it was completely insulated (the outer surface temperature of the copper heater was 500–800 °C). 

The experimental process was as follows: The vacuum pump connected to the valve at the bottom of the apparatus was operated to an inside vacuum of 0.02 bar, and then the valve was closed. Subsequently, the valve installed at the top of the apparatus was opened, and a specific amount of working fluid was injected (filling ratios (FRs) of 0.25, 0.5, and 0.75). Subsequently, power was applied to the heater using the power supply. The input power was increased from 100 to 800 W at 100 W intervals. Each input power was supplied for a sufficient time (approximately 45 min) to reach the steady state, and temperature data were collected at 1 s intervals. Subsequently, the BHTC and total thermal resistance were calculated using Equations (3) and (4):

(2) $$Q_{in}=V\cdot I,$$

where $Q_{in}$ was calculated using the data record voltage, V, and current, I.

(3) $$h_{e}=\frac{Q_{in}}{A_{e,i}\left({\bar{T}}_{e,i}-{\bar{T}}_{sat}\right)},$$

where ${\bar{T}}_{e,5}$, ${\bar{T}}_{e,6}$, and ${\bar{T}}_{e,7}$ refer to the average value of $T_{e,5}$, $T_{e,6}$, and $T_{e,7}$, respectively, for 500 s; ${\bar{T}}_{e,i}$ is the average value of ${\bar{T}}_{e,5}$, ${\bar{T}}_{e,6}$, and ${\bar{T}}_{e,7}$; ${\bar{T}}_{sat}$ refers to the average value of $T_{sat}$ for 500 s (the saturation temperature of the working fluid based on the saturation pressure inside the channel obtained through the pressure transducer).

(4) $$R_{tot}=\frac{{\bar{T}}_{e,i}-{\bar{T}}_{c,i}}{Q_{in}},$$

where ${\bar{T}}_{e,i}$ and ${\bar{T}}_{c,i}$ refer to the average inner surface temperature of the evaporator and condenser channel, respectively.

Table 2 shows the uncertainty of each measurement equipment used in the experiment. Table 3 shows the uncertainty of each experimental result obtained using the uncertainty calculation formula, Equation (5) [24]. The maximum uncertainties of the input heat and total thermal resistance were 0.28% and 10.67%, respectively, indicating errors of within 15%. The uncertainty of the BHTC was primarily less than 15%. However, two points indicated uncertainties exceeding 15%; hence, their BHTC data were discarded.

(5) $$\delta f=\sqrt{\overset{n}{\underset{i=1}{\sum }}{\left(\frac{\partial f}{\partial x_{i}}\delta x_{i}\right)}^{2}}$$

## 3. Result and Discussion

### 3.1. Geyser Phenomenon

**Figure 7:**
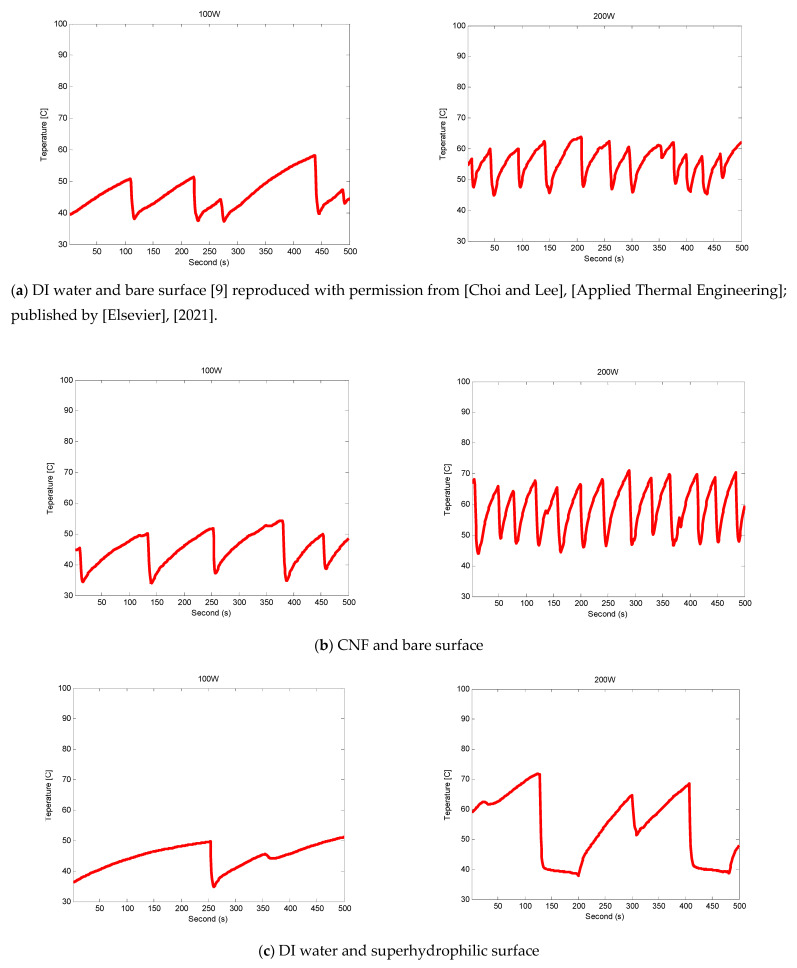

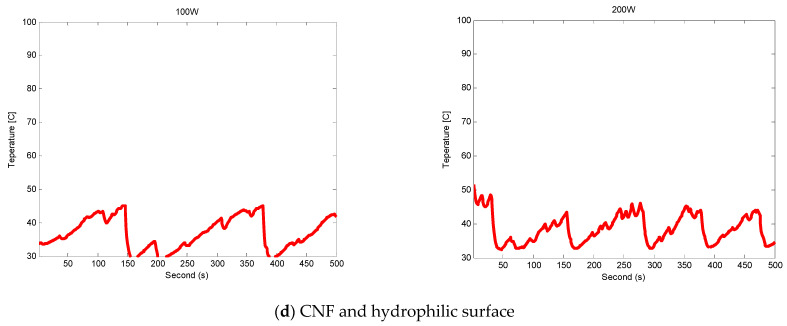
Evaporator wall temperature (T7) variations with time at FR 0.25.

Figure 7a–d show the inner surface temperature of the evaporator channel at FR 0.25 based on the working fluid (DI water and CNF fluid) and surface condition, respectively. At 100 W input power, all cases shown in Figure 7a–d indicated the Geyser phenomenon, in which the confinement effect repeated, and the temperature graph showed large amplitudes and long oscillation periods. In particular, in case (c), the oscillation period was significantly longer. At 200 W input power, cases (a) and (b) indicated the fast Geyser phenomenon, as mentioned by Choi and Lee [9], where the amplitude was the same as that of the Geyser phenomenon, but the period was shorter. However, in case (c), the temperature did not increase immediately; instead, it delayed slightly and then increased. This is because, in the FR 0.25 experiment, thin-film evaporation was more dominant than pool boiling on the inner wall of the channel, and the hydrophilic surface rewetted faster compared with the bare surface. In case (d), the period was reduced again and the amplitude was reduced significantly compared with case (c) because of the fast heat transfer due to the CNF.

The BHTC and total thermal resistance can be calculated using the temperature difference between the average temperature of the evaporator’s inner surface and the internal saturation temperature. At 100 W input power, the temperature differences of cases (a)–(d) were 13.01 °C, 7.58 °C, 3.75 °C, and 2.15 °C, respectively. At 200 W input power, the temperature differences of cases (a)–(d) were 21.60 °C, 13.45 °C, 10.53 °C, and 4.81 °C, respectively. The temperature difference decreased gradually from cases (a) to (d); hence, the highest BHTC and lowest total thermal resistance are shown in (d).

### 3.2. Hydrophilic Surface Modification

**Figure 8:**
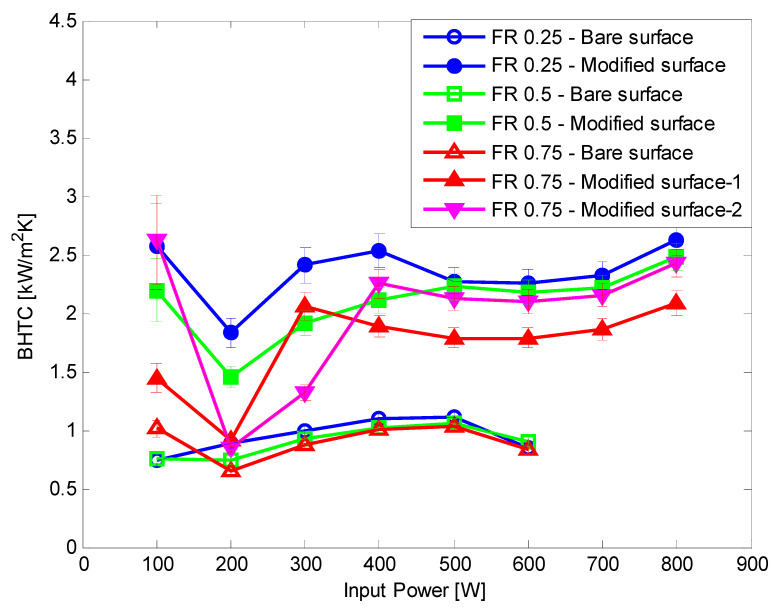
Comparison of the boiling heat transfer coefficient of bare and hydrophilic surfaces.

**Figure 9:**
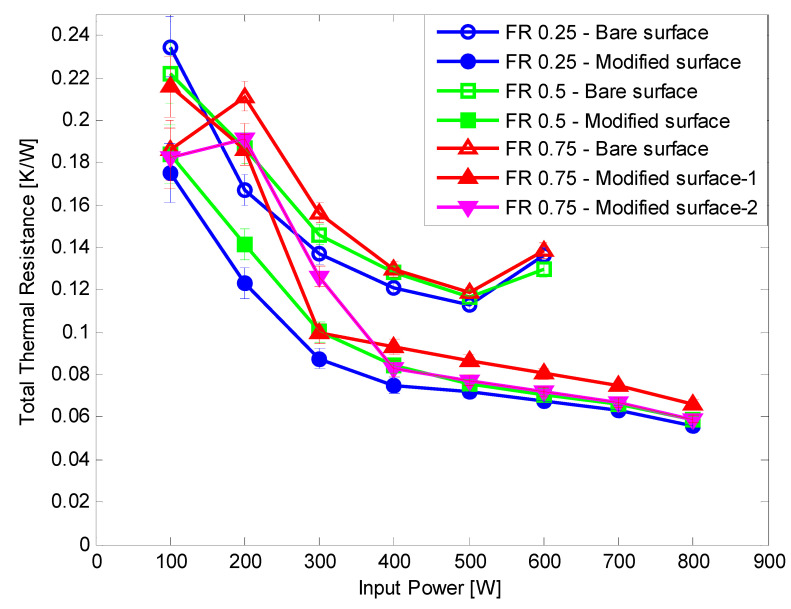
Comparison of the total thermal resistance of bare and hydrophilic surfaces.

Hydrophilic surface modification was applied to the TPCT evaporator section. In all the hydrophilic surface modification experiments, dry out did not occur up to an input power of 800 W. When surface modification was not applied, dry out occurred at 700 W. Hence, the CHF was improved by at least 14.3% compared with the bare tube case. At all FRs, the BHTC improved by at 31.09–246.87%, and the average enhancement rates were 145.75%, 104.66%, 75.55%, and 93.81% at FR 0.25, 0.5, 0.75 (for test 1), and 0.75 (for test 2), respectively (Figure 8). The total thermal resistance of all FRs reduced (Figure 9), showing an average reduction rate of 32.39%, 31.21%, 17.5%, and 20.22% at FR 0.25, 0.5, 0.75 (for test 1), and 0.75 (for test 2), respectively. The enhancement in the heat transfer performance was consistent with the basic theory that the evaporation performance of fluids on hydrophilic surfaces is better than that on bare surfaces. Furthermore, the CHF delayed due to the improved evaporation performance. In FR 0.75 test 1 and 0.75 test 2, the BHTC decreased rapidly at 200 W. It appeared that the pool boiling was dominant at 100 W due to the large amount of working fluid, and the Geyser phenomenon appeared at 200 W. In the first experiment, when going from 200 W to 300 W, the boiling heat transfer coefficient increased sharply, and after 300 W, the boiling heat transfer coefficient decreased slightly and then increased. In the second experiment, the boiling heat transfer coefficient increased relatively slowly between 200 W and 400 W, and after 400 W, the boiling heat transfer coefficient decreased slightly and then increased. Practically, it is difficult to reproduce 100% of the same phenomenon during repeated experiments because very complex heat and fluid flow occur inside the TPCT. Therefore, a slight difference occurs in the rising section of the boiling heat transfer coefficient in the first and second experiments, resulting in a 300 W point result of the first experiment appearing to be higher than those of the second experiment. In the case of the bare surface, BHTC rapidly dropped at 600 W of input power. This is because the temperature of the evaporator inner surface increased significantly because it was just before dry-out occurred at 700 W.

### 3.3. CNF and Hydrophilic Surface Modification

**Figure 10:**
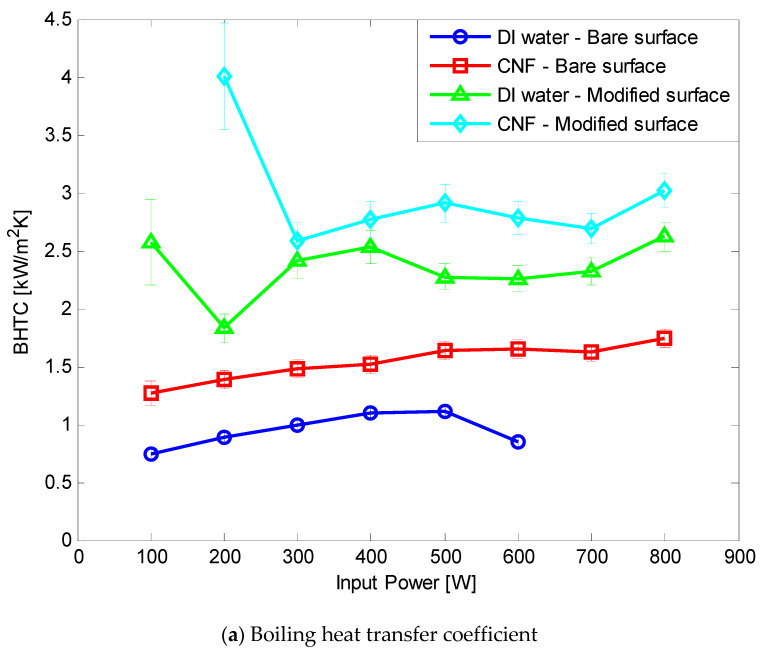

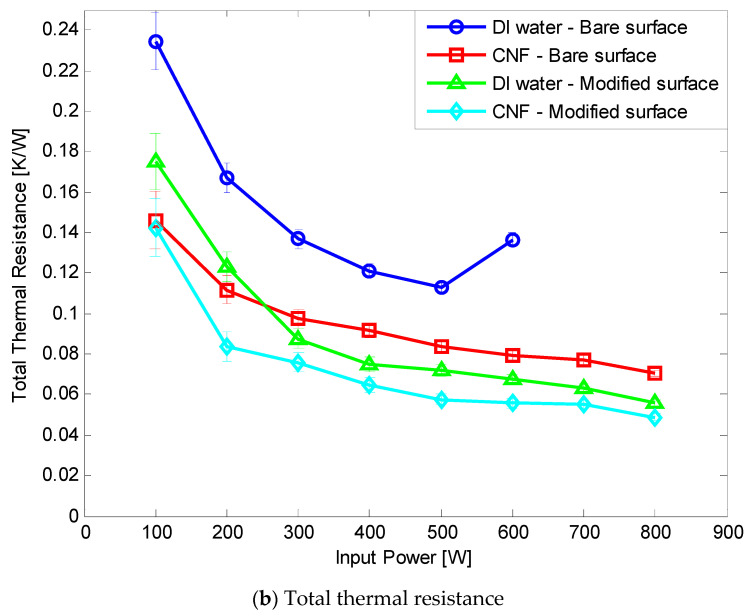
Comparison of experiment result at FR 0.25.

**Figure 11:**
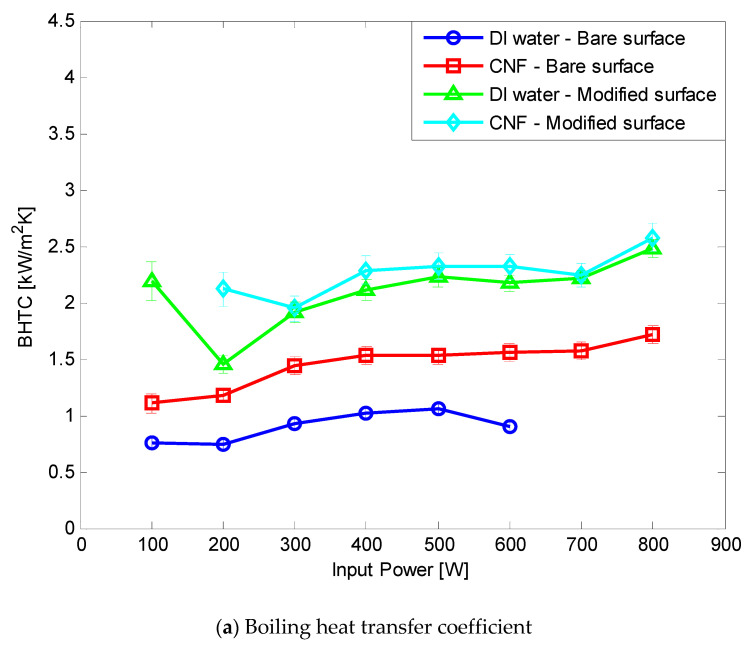

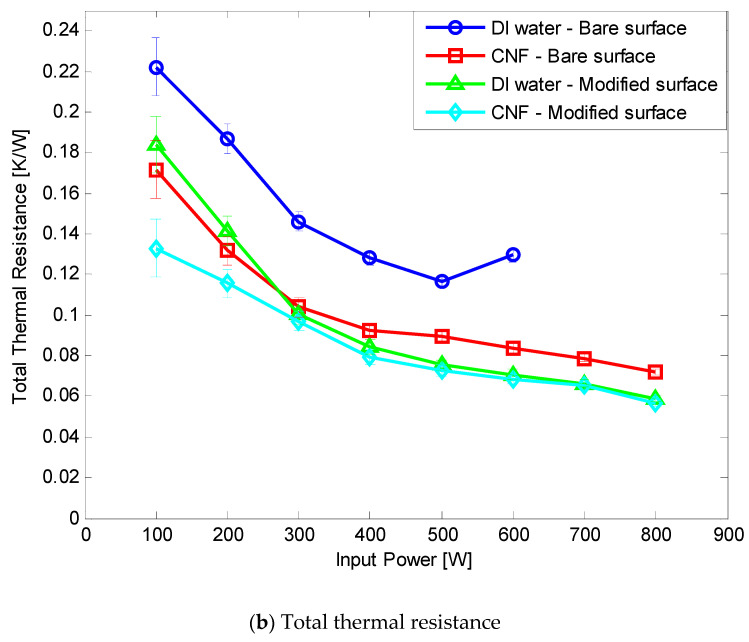
Comparison of experimental results at FR 0.5.

**Figure 12:**
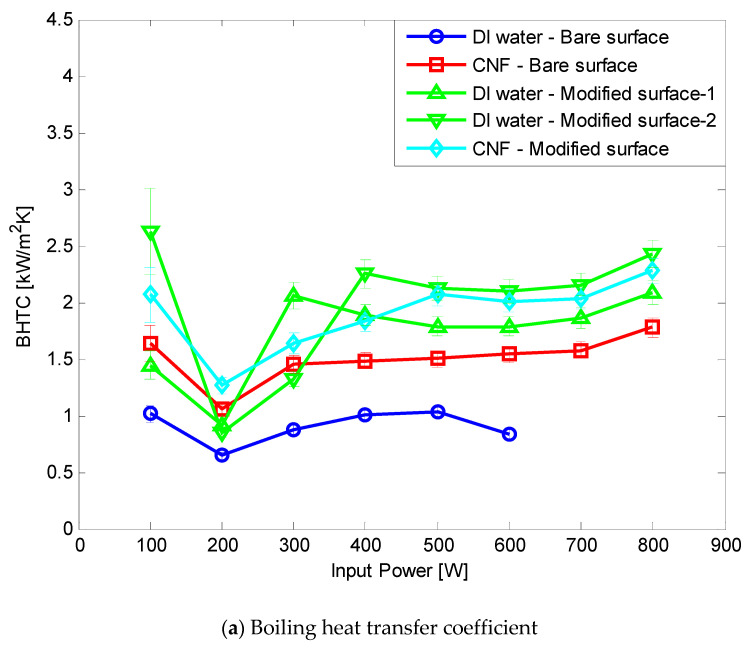

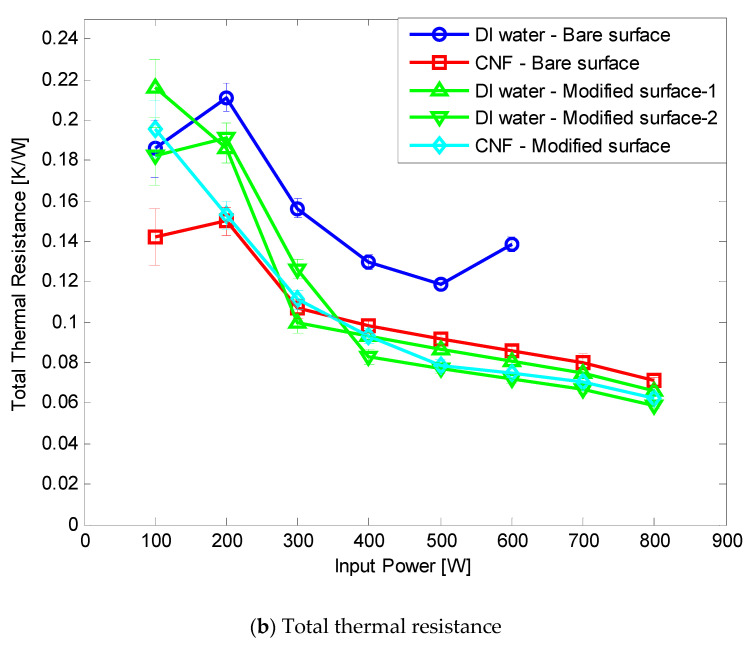
Comparison of the experimental results at FR 0.75.

Both CNF fluid and hydrophilic surface modification were applied to the TPCT. At all FRs of 0.25, 0.5, and 0.75, the CHF improved by more than 14.3% compared with the DI water and bare surface. The BHTC improved by up to 348.85% by 205.33%, 134.69%, and 93.55% on average at FRs of 0.25, 0.5, and 0.75, respectively (Figure 10, Figure 11 and Figure 12). The total thermal resistance showed average reduction rates of 47.51%, 36.69%, and 22.56% at FRs of 0.25, 0.5, and 0.75, respectively (Figure 10, Figure 11 and Figure 12). This was the best result because the effect of the rapid generation of small-sized bubbles due to the CNF working fluid [9] and the result of Section 3.2 were combined. In FR 0.5 and 0.75, the BHTC decreased rapidly at 200 W. It appeared that the pool boiling was dominant at 100 W due to the large amount of working fluid, and the Geyser phenomenon appeared at 200 W. When the Gayser phenomenon occurs, a thin liquid film is formed on the inner surface of the boiling part of the TPCT, and heat transfer occurs through evaporation. When hydrophilic surface modification is applied, water supply to the evaporation surface becomes smooth due to the enhancement of the capillary ability of the hydrophilic surface, and thus evaporation heat transfer improves. In addition, CNF basically has a high hydration ability (water-containing ability). As a result, it is estimated that when CNF is applied together, the evaporation heat transfer is improved by additionally supplying water as CNF remains on the evaporation surface. After the experiments, no aggregation and sedimentation of the CNF were observed.

**Figure 13:**
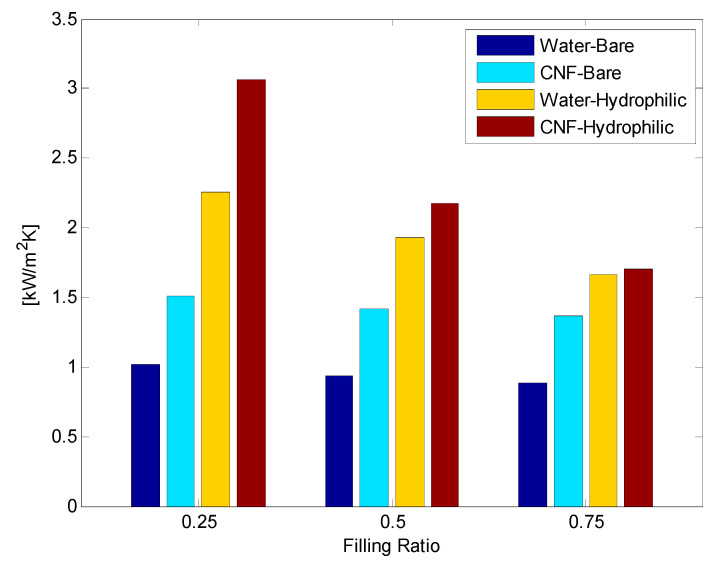
Comparison of boiling heat transfer coefficients.

Figure 13 shows a comparison of the BHTC based on each variable. As shown, the higher the FR, the smaller the enhancement rate due to the surface modification. At low FRs, film evaporation was dominant. However, at high FRs, the ratio of pool boiling increased slightly due to the large amount of working fluid. Therefore, at low FRs, the inner surface was dry and then rewetted, and the hydrophilic surface rewetted faster than the bare surface. Meanwhile, the effect of surface modification was insignificant at high FRs.

**Figure 14:**
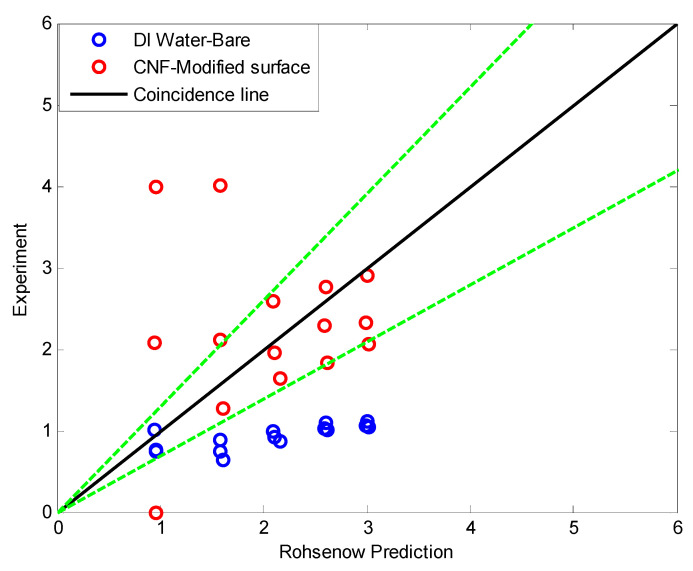
Comparison of boiling heat transfer coefficients with the Rohsenow correlation.

Figure 14 shows a comparison of BHTC with Rohsenow’s pool boiling theoretical results [25]. Before applying the CNF and the surface modification, the BHTC of this study was much lower than that of Rohsenow’s theoretical results because of the Geyser phenomenon. However, after applying the CNF and surface modification, the BHTC showed similar levels. Additionally, Table 4 shows a comparison of the BHTC obtained in this study with those of Noie [26] and Baojin et al. [27], whose Co was 0.108 and 0.120, respectively. Noie [26] and Baojin et al. used water as the working fluid, and the surface modification was not applied. Before applying the CNF and the surface modification, the BHTC of this study was much lower than those of Noie and Baojin et al. because of the Geyser phenomenon. However, after applying the CNF and surface modification, the BHTC showed similar levels. This might have maintained the heat transfer efficiency of the TPCT while increasing Co by reducing the diameter of the TPCT; this is expected to be applicable to applications requiring more compact TPCTs in the future.

## 4. Conclusions

The confinement effect occurring in a TPCT with Co = 0.245 was observed. The confinement effect degrades the heat transfer performance of the device. CNF was applied as the working fluid, and hydrophilic surface modification was applied to the inner surface of the evaporator to improve the heat transfer performance of the system. 

The findings of this study are as follows.

(1) When compared to the water and bare surface case, it was revealed that the CHF improved by at least 14.3% at all FRs (0.25, 0.5, and 0.75).
(2) The BHTC improved by up to 348.5%, whereas it improved by 205.33%, 134.69%, and 93.55% on average at FRs of 0.25, 0.5, and 0.75, respectively. The total thermal resistance showed average reduction rates of 47.51%, 36.69%, and 22.56% at FRs of 0.25, 0.5, and 0.75, respectively.
(3) In addition, because the aggregation and sedimentation of CNFs did not occur even after repeated experiments over a significant time period, it can be concluded that CNF mitigated the stability problem of the existing inorganic nanoparticles.

In order to collect a larger amount of solar heat in the same area, there is a method of reducing the diameter of TPCT and increasing the device number to increase the heat transfer area. Although the heat transfer performance decreased due to the confinement effect caused by reducing the diameter beyond the limit point, the heat transfer efficiency and the operating limit could be improved by using surface modification and working fluid. Through this method, it is expected that solar heat collection efficiency can be further increased.

In the future, the experimental apparatus should be improved to enable an input power exceeding 800 W to verify the CHF. In addition, by applying hydrophobic surface modification to the inner surface of the condenser, not only the overall thermal resistance, but also the effect of the confinement effect are expected to decrease.

## Figures and Tables

**Table 1:** Properties of the 0.5 wt% cellulose nanofiber (CNF) fluid and de-ionized water.

Property	CNF (0.5 w.t%, 25 °C)	De-Ionized Water (25 °C)
pH	7.42	7
Conductivity [mS/cm]	1.658	0.0005
$\mathrm{Density}\;[g/{\mathrm{cm}}^{3}$]	0.9984	0.9970
Viscosity [N−s/m2^]	0.0089	0.00089
Fiber Width [nm]	1–20	-
Zeta Potential [mV]	−92.83	−30~−40

**Table 2:** Uncertainty of measuring device.

Measurement	Device	Uncertainty	Range
Wall Temperature	Thermocouple (K-type, Omega)	±0.15 °C	0–700 °C
Pressure	Pressure transducer (PSHJ1000TCTJ, Sensys)	±0.15%	0–100 kPa (Abs.)
Voltage & Current	Power supply (N8953A, Keysight)	±0.2%	0–200 V, 0–75 A
Coolant Temperature	Chiller (GR-C-00050A, Busung)	±0.15 °C	−10–30 °C
Coolant Flow Rate	Flowmeter (Yuyu inst.)	±1%	0–0.7 kg/s
Thermal Power	Copper heater (Woori heater)	±0.2%	0–800 W

**Table 3:** Uncertainty of experimental result.

Result	Symbol	Uncertainty
Input Power [W]	$Q_{in}$	0.28%
$\mathrm{Boiling}\;\mathrm{Heat}\;\mathrm{Transfer}\;\mathrm{Coefficient}\;[\mathrm{kW}/m^{2}$]	$h_{e}$	4.68–14.46%
Total Thermal Resistance [K/W]	$R_{tot}$	2.39–10.67%

**Table 4:** Comparison with other studies.

200–500 W	Le/Lc (mm)	Di (mm)	Co	FR	BHTC (W/m^2K)
This study with water and bare tube	300/350	11	0.245	0.25	893–1114
				0.5	746–1055
				0.75	649–1036
This study with CNF and surface modification				0.25	2581–4008
				0.5	1950–2322
				0.75	1266–2066
Noie [26]	314/380	25	0.108	0.3	880–2250
				0.6	1075–3210
Baojin et al. [27]	350/350	22.5	0.120	0.36	3650–4710

## Nomenclature

*A*	$\mathrm{Surface}\;\mathrm{area}\;\left[m^{2}\right]$
*Co*	Confinement number
*D*	Diameter [m]
*g*	$\mathrm{Acceleration}\;\mathrm{of}\;\mathrm{gravity}\;\left[m/s^{2}\right]$
*I*	Current [A]
*L*	Length [m]
*p*	Pressure transducer
*Q*	Heat transfer rate [W]
*R*	Thermal resistance [K/W]
*Re*	Reynolds number
*T*	Temperature [℃]
*V*	Voltage [V]
*Greek symbols*	
$\lambda_{C}$	Characteristic bubble length [m]
ρ	$\mathrm{Density}\;\left[\mathrm{kg}/m^{3}\right]$
σ	Surface tension [N/m]
*Subscript*	
*c*	Condenser section
*copper*	Copper
*e*	Evaporator section
*i*	Inner
*in*	Input
*l*	Liquid
*sat*	Saturation
*tot*	Total
*v*	Vapor
*Abbreviations*	
BHTC	Boiling heat transfer coefficient
CHF	Critical heat flux
CNF	Cellulose nanofiber
DI	De-ionized
FR	Filling ratio
TPCT	Two-phase closed thermosyphon
